# Mass Spectrometry–Based Applications of Spheroids in Cancer Biology

**DOI:** 10.1146/annurev-anchem-061424-090547

**Published:** 2025-01-14

**Authors:** Brian D. Fries, Amanda B. Hummon

**Affiliations:** 1 Department of Chemistry and Biochemistry, The Ohio State University, Columbus, Ohio, USA; 2 Comprehensive Cancer Center, The Ohio State University, Columbus, Ohio, USA

**Keywords:** cancer, spheroids, three-dimensional cell culture, mass spectrometry, proteomics, mass spectrometry imaging

## Abstract

The use of cell culture techniques to model human disease is an indispensable tool that has helped improve the health and well-being of the world. Monolayer cultures have most often been used for biomedical research, although not accurately recapitulating an in vivo human tumor. Tumor spheroids are a form of three-dimensional cell culture that better mimics an avascularized human tumor through their cell–cell contacts in all directions, development of various chemical gradients, and distinct populations of cells found within the spheroid. In this review, we highlight how mass spectrometry has propelled the utility of the spheroid model to understand cancer biology. We discuss how mass spectrometry imaging can be utilized to determine the penetration efficiency of various chemotherapeutics, how proteomics can be used to understand the biology in the various layers of a spheroid, and how metabolomics and lipidomics are used to elucidate how various spheroids behave toward chemotherapeutics.

## INTRODUCTION

Cell culture models are an indispensable tool utilized by researchers in the biomedical sciences. These models have been used to manufacture vaccines for diseases such as polio and influenza (1). Cell culture techniques are also widely used in the pharmaceutical industry to determine the efficacy of many therapeutics. The International Space Station has even used cell culture models to help understand how microgravity affects the growth of cells compared to their earthbound counterparts (2).

Two-dimensional (2D) cell cultures have been the most widely used model, where cells are grown in a monolayer on tissue culture plates or in suspension. 2D cell cultures benefit from being cost-effective, fast to grow, and much easier to manipulate for automated procedures. However, 2D cell culture lacks the complexity typically found within an animal tissue, such as cell–cell contacts in three-dimensional (3D) dimensions, differing cell populations, and varying amounts of nutrients. 3D cell culture models—such as multicellular tumor spheroids (MCTSs, commonly referred to as spheroids) and organoids—offer an attractive alternative to bridge both 2D cell cultures and animal models. Spheroids better mimic an in vivo human tumor, generating distinct layers with different populations of cells and allowing for the penetration efficiency of a therapeutic to be evaluated (3, 4). Spheroids are cost-effective and can be grown quickly in large numbers for high-throughput studies using a 96- or 384-well plate (5). Many analytical techniques can be used to analyze spheroids, such as Raman spectroscopy (6, 7), electrochemistry-based methods (8, 9), and even nuclear magnetic resonance (NMR) spectroscopy of whole spheroids (10, 11). In this review, we primarily focus on the development of the spheroid model system and how the spheroid model has been further characterized by mass spectrometry (MS)-based techniques.

## SPHEROIDS

**Figure 1:**
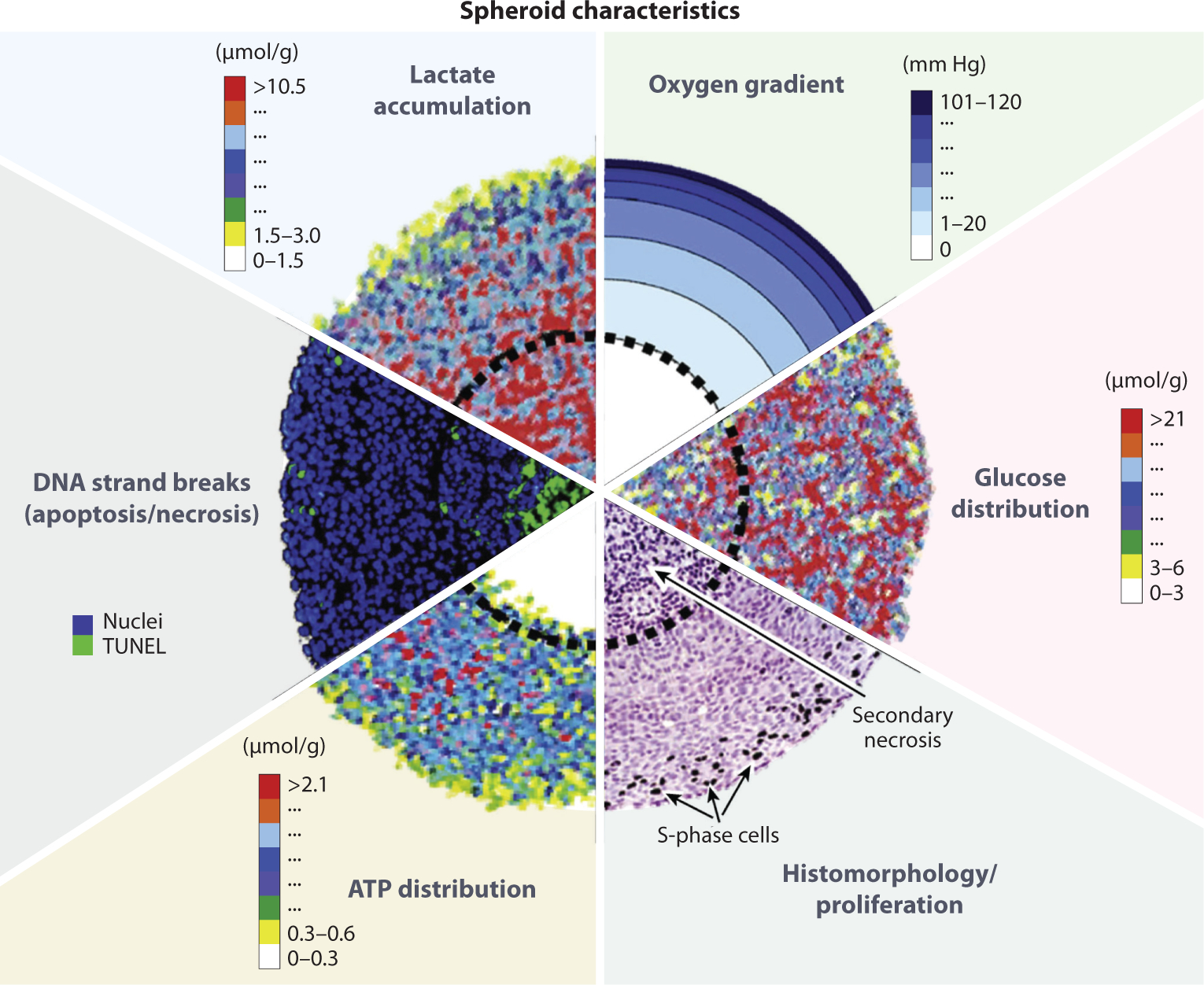
Chemical and nutritional gradients found within a spheroid. The gradual decrease of oxygen and the accumulation of lactate in the core of the spheroid allow for the generation of the central necrotic core (the core edge is indicated by a *dashed line*). Staining of cells also shows that the outer layers of the spheroid contain cells that are actively dividing. Figure adapted with permission from Reference 35; copyright 2021 American Society for Biochemistry and Molecular Biology.

**Figure 2:**
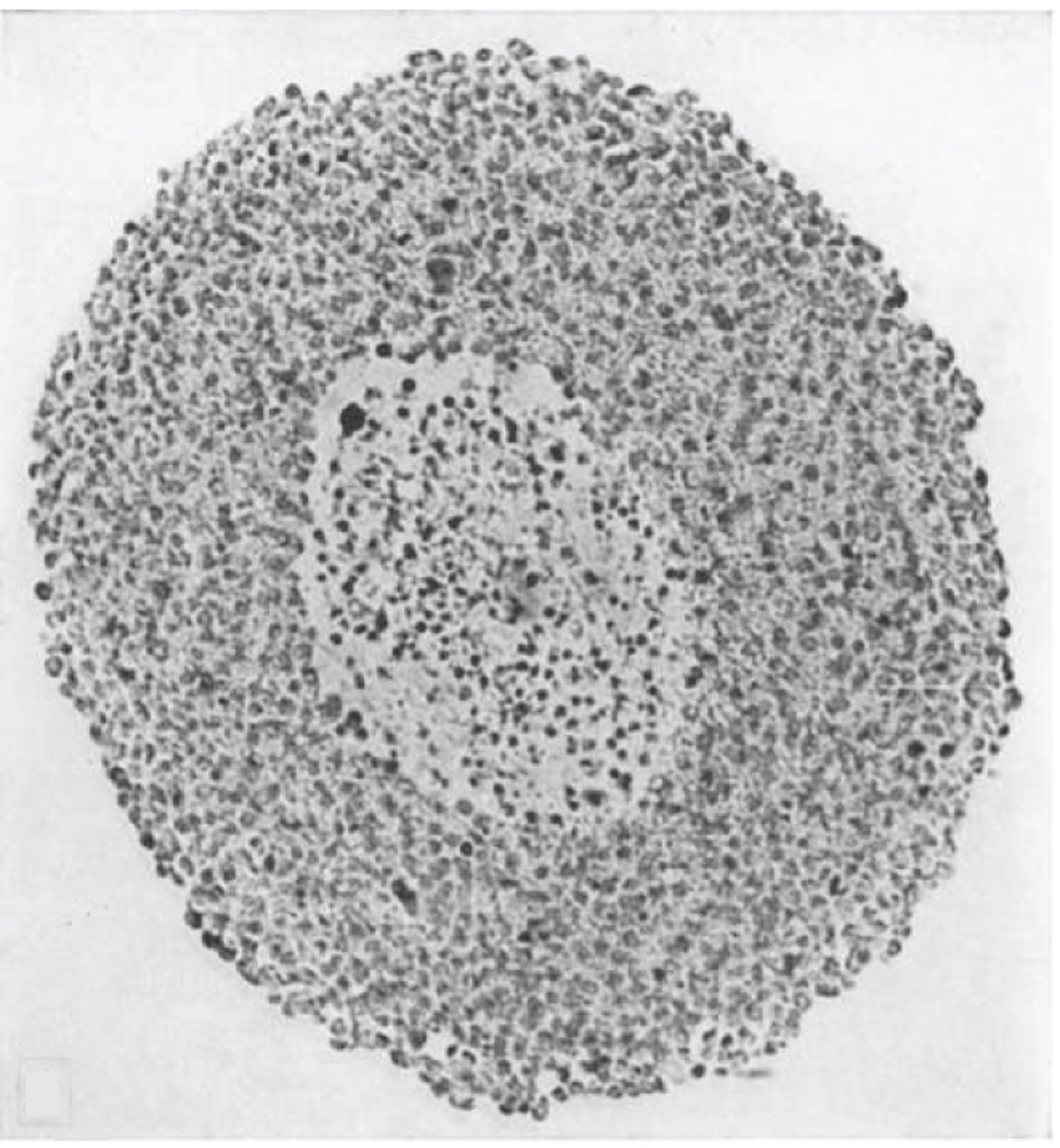
Section of a V79 spheroid stained with hematoxylin and eosin (H&E) showing a central necrosis along with proliferating outer cells. The spheroid was grown for 24 days in a suspension culture and was 450 μm in diameter. Figure adapted with permission from Reference 12; copyright 1971 Oxford University Press.

Spheroids are a multicellular 3D cell culture model that develops various gradients in nutrient levels, oxygen availability, pH, and cellular populations (Figure 1). In 1971, Robert Sutherland and coworkers grew some of the first spheroids from Chinese hamster V79 cells (12). They added cells to a flask and kept them suspended in media, allowing them to aggregate together and form spheroids. The authors noted that these spheroids demonstrated exponential growth for 7 days and then started a slower growth rate after day 9 (12). Hematoxylin and eosin (H&E) staining of sectioned spheroids showed three distinct zones within the spheroid: an outer proliferating zone, an intermediate zone with larger cells along with more spread-out nuclei staining, and a central necrotic zone (12) (Figure 2). To confirm these layers, the spheroids were labeled with ^3^H-thymidine, and the autoradiograph showed more labeled cells in the outer region, poorly labeled cells in the intermediary region, and unlabeled cells in the center of the spheroid (12). The generation of these different regions was similar to the development of an avascular human tumor, increasing interest in using this model to better mimic in vivo human tumors. Resistance to radiation treatment in a spheroid was shown first by F.W. Hetzel and colleagues in 1973 (13). These authors showed that the outer cells of the spheroid are negatively afflicted by radiation, whereas the central necrotic cells do not feel the same effects of radiotherapy. It was then shown in 1974 that altering the metabolism of the spheroid can start to increase the therapeutic potential of the ionizing radiation (14). These initial spheroids were further characterized with microelectrodes to measure the partial pressure of oxygen (*p*O_2_) amount in the different regions of the spheroid, showing that the amount of oxygen rapidly decreases from the outside of the spheroid to the core (15–17).

Spheroids can be grown from many different human and animal cell lines using a diverse set of methods (18). Oftentimes, cells are seeded onto a nonadherent surface, such as low-adhesion 96-well plates or on a thin layer of a nonadherent surface, such as poly-HEMA or agarose. The cells then aggregate and adhere to each other, thus forming a spheroid. This method—also termed the liquid overlay method—was developed by Ivascu & Kubbies in 2006 (19), where they seeded eight different breast cancer cell lines at 5,000 cells/well in poly-HEMA-coated 96-well plates and allowed them to form into spheroids. Only three cell lines successfully formed spheroids: MCF7, T47D, and MDA-MB-435. Another common way to make spheroids is via the hanging drop method, where droplets of suspended cells are placed on the lid of a petri dish and inverted, causing the cells to fall to the bottom of the liquid droplet and adhere together (20). The hanging drop method allows for a more even distribution of nutrients and therapeutics to be obtained as the spheroids are moved around during growth. Newer methods of preparing spheroids are constantly being developed, such as using magnetic nanoparticle-incorporated cells to form spheroids upon exposure to a concentrated magnetic field or using microfabricated stencils to form many spheroids at once with very consistent sizes (21, 22).

Spheroids share many similarities with another common 3D cell culture model: organoids. Organoids are 3D cell culture models derived from stem cells (23). As cells are growing within the organoid, they will differentiate and generate structures similar to an organ (e.g., intestinal organoids will generate crypts and villi, mimicking the shape and function of a small intestine). Organoids benefit from being recently derived from an actual patient and can be used for personalized medicine purposes. Unlike spheroids, organoids are not grown with immortalized cells and therefore require specialized cell culture techniques and expensive materials, such as matrigel. Spheroids thus allow for a more cost-effective alternative to determine the efficacy of a therapeutic prior to investing time and money into a more expensive cell culture technique.

A spheroid, as originally defined by Sutherland, is a collection of cells adhered together and grown for roughly 12–14 days. This amount of time is necessary to allow the radially symmetric chemical gradients to properly develop and thus lead to the creation of the pathophysiological gradients within a spheroid (12). Seeding cells in a nonadherent environment will cause them to aggregate and, without ample time to grow, will generate a multicellular aggregate. Many recent studies have featured these cellular aggregates as samples of interest, often growing the aggregates for 4–7 days instead of the full 12- to 14-day growing period described by Sutherland et al. (12). While these multicellular aggregates are an intriguing model that can be generated rapidly, they do not meet the specifications of the original spheroid model developed by Sutherland. A multicellular aggregate grown for less than 12 days does not have time to develop the radially symmetric chemical or pathophysiological gradients. Without these critical gradients, these cellular aggregates do not have the same relevancy as a tumor model.

## DRUG TESTING AND MASS SPECTROMETRY IMAGING

One of the most valuable applications of the spheroid model system is in the realm of chemotherapeutic testing (Table 1). Since 1938, every therapeutic approved by the US Food and Drug Administration (FDA) has been required to first be tested in an animal model prior to testing in humans through the Federal Food, Drug, and Cosmetic Act. However, this has recently been changed by the passing of the FDA Modernization Act 2.0 in 2022, removing the requirement of testing in animal models and instead approving the use of more complex cell culture systems (24). The spheroid model system allows for the penetration efficiency of therapeutics to be evaluated, as well as to assess the response of genes that are otherwise inactivated in 2D cultures (25). One of the first therapeutics to show success in 2D cultures but failure in 3D cultures was doxorubicin (brand name Adriamycin^®^) (26). Recently, it has been shown that if fatty acid synthase inhibition had been first demonstrated in spheroids, failed animal testing would not have occurred (27, 28).

Many other therapeutics have been shown to be ineffective in spheroids, such as methotrexate in osteosarcoma spheroids (29), verapamil sensitization to Adriamycin in V79 spheroids (30), as well as increased drug resistance of the MCF7 cell lines. Poor penetration of paclitaxel in DLD-1 spheroids (31) has also been shown. An intensive study by Świerczewska et al. (32) showed the various responses of cisplatin, methotrexate, doxorubicin, vincristine, paclitaxel, and topotecan in seven different ovarian cancer cell lines grown both in monolayers and as spheroids. The authors observed that spheroids were consistently more resistant to the chemotherapeutics compared to monolayer cultures and determined that extracellular matrix (ECM) genes are the probable cause for the increased resistance. The expression of the *LOX* gene, encoding the protein lysyl oxidase, was elevated in spheroids compared to monolayer cultures (32). Lysyl oxidase converts lysine residues into allysine, which then forms cross-links in ECM proteins, thus potentially contributing to chemoresistance mechanisms.

Monitoring a therapeutic as it travels farther in the spheroid can be challenging, as most analytical techniques require the therapeutic to have unique spectral features that do not overlap with many biomolecules. Mass spectrometry imaging (MSI) is a powerful label-free technique that can be used to spatially resolve the position of small molecules within a biological sample (33, 34). MSI works by rostering a laser across a thin section of tissue on a conductive slide [typically indium tin oxide (ITO) slides] and collecting a full mass spectrum at each pixel. By performing this technique, various mass-to-charge (*m*/*z*) values can be spatially visualized and annotated with targeted techniques. Typically, small molecules such as therapeutics are visualized using MSI, but lipids and metabolites are often mapped using MSI to perform spatial lipidomics and metabolomics. The combination of MSI and spheroids allows the penetration efficiency of a therapeutic to be fully evaluated using a label-free technique (35).

**Figure 3:**
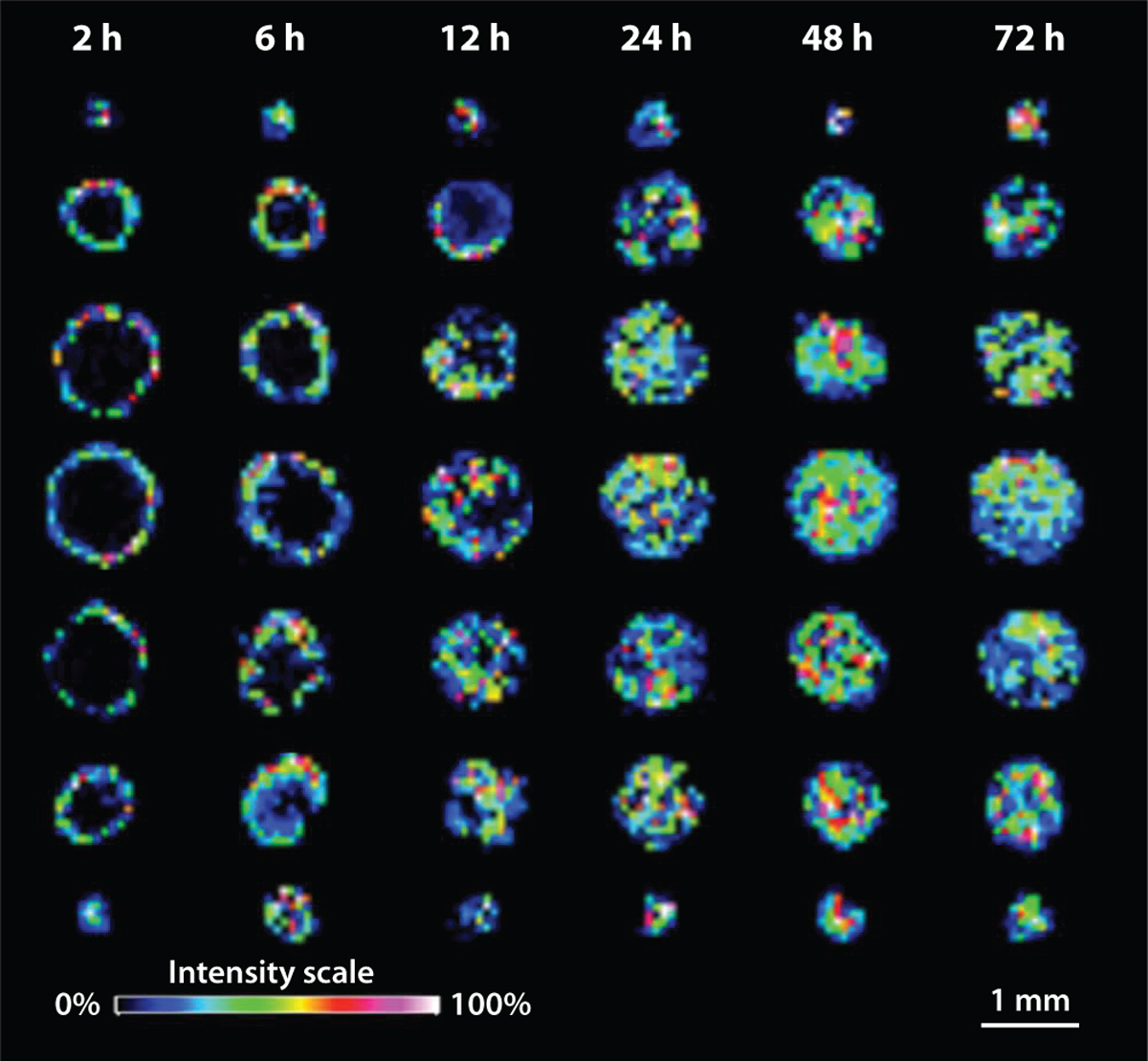
Time course MSI data from HCT 116 spheroids treated with irinotecan (*m*/*z* 587) showing penetration throughout the spheroid. The intensity scale refers to the color-coded representation of the signal strength detected at each pixel in an image, with pink indicating the highest signal intensity and blue showing the lowest. Figure adapted with permission from Reference 4; copyright 2013 American Chemical Society. Abbreviations: MSI, mass spectrometry imaging; *m*/*z*, mass-to-charge.

The Hummon lab was one of the first to profile various lipids and peptides within HCT 116 spheroids using MSI (36). With this method, certain ions were found to be either spread throughout the entirety of the spheroid or localized solely to the central core region of the spheroid. Matrix-assisted laser desorption ionization (MALDI) MSI was then applied to HCT 116 spheroids exposed to irinotecan, a prodrug that gets metabolized to the active form, SN-38, and inhibits topoisomerase I. Irinotecan remained in the outer edges of the spheroid after 2 h and 6 h, with further penetration into the middle regions of the spheroid after 12 h and fully penetrated throughout the spheroid after 24 h (4) (Figure 3). Analysis of SN-38 (*m*/*z* 569) in the MALDI MSI data set showed higher abundance in the outer regions of the spheroid and very low abundance in the core of the spheroid. This is attributed to the higher metabolism of the outer proliferating cells converting more of the prodrug to the active form.

Small molecules are some of the most common analytes imaged using MSI. Recently, Chen et al. (37) showed how lipid metabolites are altered in MSI data after A549 spheroids were exposed to hydroxychloroquine (HCQ). HCQ was observed to increase in intensity from 2 h to 48 h, largely localizing to the core of the spheroid, potentially due to the lower metabolism of HCQ in the core of the spheroid (37). In conjunction with untargeted liquid chromatography tandem mass spectrometry (LC-MS/MS) lipidomics, the identification and spatial distribution of altered lipids were analyzed. The MSI data showed that phosphatidylinositol (PI) [(38:3)-H]^−^ (*m*/*z* 887.56) was elevated in the outer region of HCQ-treated spheroids but was downregulated in the core (37). Palubeckaitė et al. (38) analyzed the spatial distribution and metabolic changes from osteosarcoma spheroid aggregates made from SAOS-2 cells exposed to doxorubicin. Doxorubicin was observed to fully penetrate into the spheroid aggregate after 6 h. Using MSI to putatively identify various metabolites, the authors showed that *S*-nitrosylglutathione increased in intensity with increasing doxorubicin exposure throughout the spheroid aggregate (38). Spheroids have also been generated from malignant ascites, which are cells from fluids within human tumors. Mittal et al. (39) generated primary spheroids from ovarian cancer patient malignant ascites and determined various lipid alterations in primary spheroids treated with and without carboplatin. MSI has also been applied to quantitatively evaluate drug penetration within a spheroid, as shown by Wang & Hummon (40) for absolute quantification of irinotecan within HCT 116 spheroids.

MALDI MSI has also been used to image much larger molecules—such as penetration of antibodies and distribution of proteins and metals—in spheroids. Liu et al. (41) imaged the monoclonal antibody therapeutic cetuximab, which is a challenge because whole antibodies approach the upper mass range limit for most MALDI mass spectrometers. This issue was alleviated by conducting an on-tissue reduction and alkylation to separate the light and heavy chains of the antibody from each other. MSI in HT-29 spheroids treated with cetuximab showed even therapeutic distribution throughout the spheroid, whereas DLD-1 spheroids had cetuximab largely accumulate in the outer proliferative layer (41). This distribution may be attributed to the DLD-1 spheroids having naturally higher levels of epidermal growth factor receptor (EGFR) expression, thus causing cetuximab to build up on the outside of the spheroid and not penetrate farther into the spheroid. Using laser ablation–inductively coupled plasma (LA-ICP)-MSI, Flint et al. (42) showed the spatial distribution of various proteins in HCC827 lung adenocarcinoma cells by mapping heavy metals bound to protein-specific antibodies that had been applied to spheroid sections. The authors observed E-cadherin and pan-cytokeratin to be located throughout the spheroid, but not present in the core, possibly due to cell breakdown from necrosis. Glut1, a marker for hypoxia, was largely located to the core of the spheroid. Overlaying the Glut1 with K_i_-67 showed distinct regions of the tumor microenvironment where there is predominant expression of K_i_-67 in the outer proliferative and middle quiescent regions but little Glut1 in these outer regions (42). The authors also used LA-ICP-MSI to map the abundance of metals in spheroids. Mg and Zn, metals that play vital roles in cell division and metabolism, were observed to be highly abundant in the outer and middle regions of the spheroid (42). Cu levels were elevated in the necrotic core, possibly as the result of upregulation of the Cu transporter ATP7A under hypoxic conditions, with Cu becoming part of the buildup of metabolic debris in the core (42).

## PROTEOMICS

Proteomics is a powerful analytical technique used to quantify many proteins within a biological system using methods such as sodium dodecyl sulfate polyacrylamide gel electrophoresis (SDS-PAGE), MALDI MS, and LC-MS/MS. Reviews of proteomics can be found elsewhere (43, 44). In a typical bottom-up LC-MS/MS proteomics experiment, tryptic peptides are separated using a C18 column and fragmented to obtain their identity. Peptide fragments are matched to in silico–generated fragments to determine the most probable peptide identity. Normalized peptide intensities from multiple peptides found within a protein are summarized together to obtain a proxy for protein abundance in a sample. Using offline enrichment methods, posttranslational modifications, such as phosphorylation and glycosylation, can also be studied to discern cellular signaling mechanisms through phosphoproteomics, ubiquitinomics, and glycoproteomics, to name a few. More information on these subdisciplines within proteomics can be found in review articles (45–48). Proteomics has been widely used for understanding protein pathways in cancer biology and has been adapted by many researchers to study cancer spheroid biology as well.

**Figure 4:**
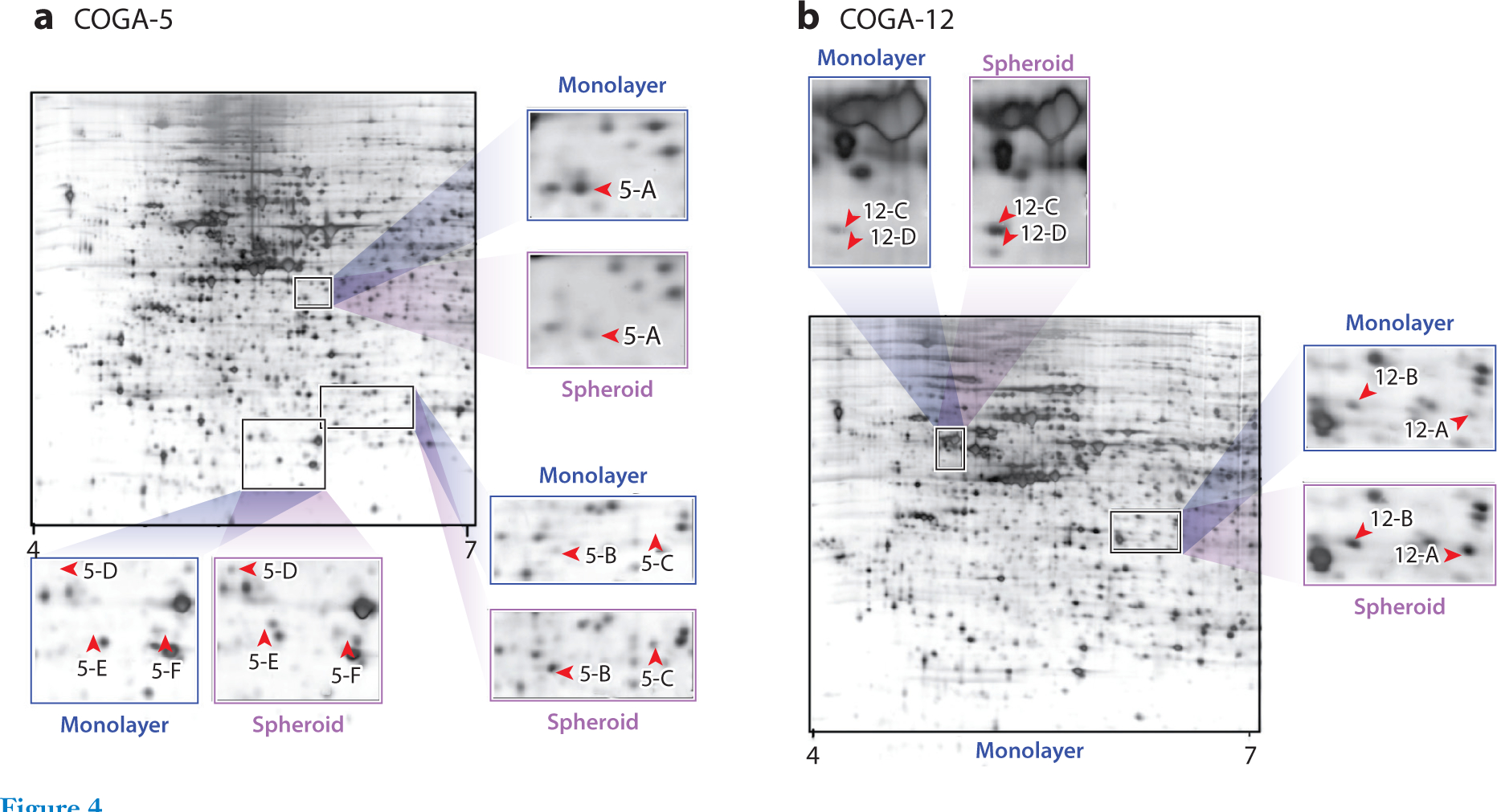
Two-dimensional (2D) gel electrophoresis of monolayer and spheroid (*a*) COGA-5 and (*b*) COGA-12 cells. Different profiles of proteins are shown through this gel, and these proteins were cut out of the gel and analyzed using mass spectrometry for identification and quantification. Figure adapted with permission from Reference 49; copyright 2007 American Chemical Society.

One of the first proteomic analyses of spheroids was conducted by Gaedtke et al. (49) in 2007. In this study, the authors compared the colon cancer cell line COGA-5 grown as spheroids and as monolayers. Using 2D gel electrophoresis with MALDI MS for peptide identification, three proteins were identified as differentially expressed in spheroids compared to monolayers: acidic calponin, 15-hydroxyprostaglandin dehydrogenase, and lamin A/C (49) (Figure 4). Lamin A/C was observed to increase as the spheroid became more compact, possibly due to increased hypoxic conditions and lack of nutrients in the spheroid. Depriving the monolayer cells of serum as well as placing them under hypoxic conditions caused the lamin A/C expression to increase (49). Then, in 2008, Kumar et al. (50) conducted a comparison of neuroblastoma (NB) cell lines grown as spheroids and monolayers using 2D gel electrophoresis fractionation followed by LC-MS/MS analysis. Metabolic proteins, such as alpha enolase, transketolase, triosephosphate isomerase, and pyruvate kinase M1/M2, were elevated in NB spheroids compared to monolayers. The authors also observed cell stress response (e.g., heat shock proteins), and structural proteins (e.g., septin 2, tubulin β-2 chain, and actin) to be elevated in spheroids (50).

An in-depth study of the proteome and phosphoproteome was conducted in 2016 by Yue et al. (51) using stable isotope labeling of amino acids in cell culture (SILAC) of HT-29 spheroids and monolayers. SILAC works by metabolically labeling cells with heavy, medium, or light isotopes of arginine and lysine and quantifying peptides based on the MS1 label intensity (52, 53). Proteins in the mitochondrial respiratory complex were upregulated in spheroids compared to monolayer cells, suggesting that spheroids were undergoing more oxidative phosphorylation. Downregulated proteins found in spheroids included minichromosome maintenance complex proteins—a complex required for genome replication—indicating that DNA synthesis is lowered in spheroids most likely because cell division is largely occurring in the thin outer proliferative layer (51). The phosphoproteomics results showed that MAPK1, PTK2, and PAK4 phosphoproteins were all downregulated in spheroids due to their slower growth rate compared to monolayer cells. Intriguingly, the phosphorylated form of mutS homolog 6 (MSH6), a protein involved in DNA repair, was downregulated in spheroids either due to the slower growth rate of spheroids or increased DNA damage resistance in spheroids. Utilizing both proteomics and phosphoproteomics, Frederick et al. (54) characterized altered signaling pathways in spheroids that had reattached to tissue culture plates. The authors observed that ROCK1 activation increases upon spheroid formation, but decreases upon spheroid re-adherence to tissue culture plates, playing a role in cell proliferation in spheroids and increased metastasis in vivo (54). Ding et al. (55) used phosphotyrosine phosphoproteomics to discern signaling differences in lapatinib-treated BT474 breast cancer spheroids. The authors observed increases in the phosphosites pY542 and pY580 in Src homology-2 (SH2) domain-containing phosphatase 2 (SHP2) in lapatinib-treated spheroids compared with control spheroids (55).

While several studies have been conducted to elucidate the phosphoproteome of spheroids, more analytical techniques and methods have been employed to study other important posttranslational modifications in cancer spheroids. Zhou et al. (56) analyzed the glycoproteome and glycocalyx of both HCT 116 and HT-29 spheroids. A twofold increase in abundance of high-mannose N-glycans in spheroids was observed when comparing the cells to their respective monolayer counterparts. *O*-sialylated glycans were also observed to be more abundant in the spheroid model than in monolayer cultures; specifically, Hex_4_HexNAc_4_ in HCT 116 and Hex_1_HexNAc_1_Fuc_1_NeuAc_1_ in HT-29 spheroids were found to be statistically significant (*p* < 0.05) when compared to the monolayers (56). Stransky et al. (57) extracted histone proteins from HepG2/C3A spheroids to examine different patterns of histone acetylation of spheroids exposed to sodium butyrate for three days and then removed until day 10. Three days after sodium butyrate exposure, the number of histone H4 peptides that contained multiple acetylations increased, and seven days after sodium butyrate exposure H4 peptide acetylation levels were back to their pretreated state, showing that the spheroids are able to recover from an inhibitor exposure (57).

A solid tumor will begin as an avascularized mass of cells. As this avascularized tumor grows, layers will develop within the tumor: a central necrotic core, a middle quiescent layer, and an outer proliferative layer (58, 59). As previously mentioned, and as shown in Figure 2, the spheroid model system develops these same distinct layers as they grow. These layers develop from the lack of nutrients, decreasing amounts of oxygen, and increasing amounts of acidic metabolic by-products from the necrotic core. The ability to discretely study these individual layers adds to the growing utility of the spheroid model for cancer research. Sutherland et al. (12) separated these layers by a process termed serial trypsinization. Serial trypsinization occurs by incubating whole spheroids with very low concentrations of trypsin with constant agitation, causing cells to slough off for collection. The trypsin is neutralized with cell culture media containing serum, and the process is repeated multiple times until the central necrotic core is left. This method has proved to have great utility, but this process is tedious and has large room for error, requiring constant pipetting steps for multiple hours to fully separate each layer for analysis. In 2012, McMahon et al. (60) used proteomics to study these distinct layers in HT-29 spheroids following serial trypsinization. These authors observed that 8 of the 12 identified glycolytic enzymes were upregulated in the core of the spheroid as well 9 enzymes involved in steroid biosynthesis. It was also observed that integrin α-1, involved in cell–cell adhesion, increased in abundance as one moved from the outer regions of the spheroid to the core. As mentioned above, Zhou et al. (56) also conducted glycomics on these distinct spheroid layers and observed elevated levels of high-mannose N-glycans in the proliferative layer and the necrotic core produced N-glycans with more complex structures than the proliferative layer.

A method to quantitatively study the proteome in the layers of a spheroid without serial trypsinization was developed by Beller et al. in 2021 (61). They utilized SILAC to progressively label different regions of the spheroid as it grows. Spheroids are grown initially for four days with a heavy SILAC label, then all of the cell culture media is replaced with a medium SILAC label, and spheroids are grown for four days only with the medium SILAC label. Lastly, all the medium SILAC media is removed and replaced with light-labeled SILAC media, and the spheroids are grown only in light media until day 12. Because spheroids grow outwardly in a radially symmetric pattern, the SILAC labels are added in progressive shells. Growing the spheroids in these three different SILAC labels allows the creation of isotopic zip codes, where each label is correlated to a distinct layer of the spheroid (61). The proteins fructose bisphosphate aldolase A and alpha enolase were more abundant in the label given to the outer layer of cells, and necrotic markers such as prohibitin-1 and prohibitin-2 were consistently more abundant in the label given to the core cells. This model was further utilized to analyze how a therapeutic, regorafenib, altered the core differently than the outer proliferating regions (62). Many pathways were altered differently in the core versus the proliferating outer layer after regorafenib inhibition, with pathways such as phosphoinositide 3-kinase and protein kinase B (PI3K/AKT) predicted to be deactivated in the outer regions and activated in the core regions of the spheroid after inhibition (62). SILAC was also used by Guo et al. (63) to understand cross talk between MDA-MB-231 breast cell cancer cells cocultured with THP-1 macrophage cells to understand how tumor-associated macrophages contribute to tumor progression. SILAC proteomics showed upregulation of Ras-related C3 botulinum toxin substrate 2 (RAC2) in cocultured spheroids. Knocking down the levels of RAC2 in the spheroids caused decreased proliferation, invasion, migration, and epithelial-mesenchymal transition (63).

As stated previously, testing of therapeutics is a vital application of spheroids for cancer research. With the ever-expanding field of MS-based omics, the distinct molecular differences can be determined for spheroids exposed to many different therapeutics. In 2013, Arai et al. (64) showed that osteosarcoma spheroid resistance to doxorubicin was attributed to cathepsin D, a protein observed to be expressed in greater abundances in spheroids compared with monolayer cultures. Lamb et al. (65) in 2014 used proteomics to identify potential drug targets in MCF7 and T47D breast cancer spheroids that would otherwise not be adequate drug targets in monolayer cultures. Mitochondrial enzymes were upregulated in spheroids compared with monolayers. Inhibitors for mitochondrial fuel uptake were then applied to spheroids, and they were highly sensitive to the inhibitors, having a 50% maximum inhibitory concentration (IC_50_) value of about 1 μM for the inhibitors (65).

The use of MS to understand the proteomic response to therapeutics in spheroids has continued to increase. Park et al. (66) used chemical proteomics along with HepG2 spheroids to show that the EGFR inhibitor ertredin causes a decrease of hypoxia-inducible factor 1 alpha (HIF-1α) in spheroids, potentially affecting a decrease in hypoxia inducible genes in spheroids. Spheroids have been used to understand gained chemoresistance to temozolomide in glioblastoma spheroids cultured from U87 cells with an artificial blood–brain barrier (67). Spheroids were particularly useful for this study, as most in vivo glioblastomas develop a hypoxic core that is lacking in many nutrients, making spheroids a very similar model system to study this disease. Using untargeted proteomics, Lam et al. (67) showed that U87 spheroids with a blood–brain barrier treated with temozolomide had upregulation of many cell cycle and cytoskeletal proteins compared to dimethyl sulfoxide (DMSO) control spheroids. Correlation of the proteomics data with data obtained from the Chinese Glioma Genome Atlas showed that patients with cell cycle genes (such as *TOP2A*, *KIF2C*, and *CDK1*) enriched had poor survival, matching proteomic observations that temozolomide-treated spheroids had upregulated cell cycle proteins (67).

## METABOLOMICS AND LIPIDOMICS

In tandem with the rise in popularity of proteomics, there has also been significant interest in studying the metabolome and lipidome of biological samples. The metabolome can show what has happened in a system and how that system has responded to an external stimulus. Metabolomics and lipidomics are ever-expanding fields and have been further reviewed: Weckwerth (68), Liu & Locasale (69), and Schmidt et al. (70) for metabolomics and Wang et al. (71), Rustam & Reid (72), and Salita et al. (73) for lipidomics. Metabolomics has amplified the field of cancer biology, for example, showing differences in the metabolism of colorectal tumors and neighboring mucosa (74), identifying lipid biomarkers for diagnosis of early-stage lung cancer (75), and gleaning alterations into amino acid, nicotinate, and purine metabolism in prostate cancer (76). Untargeted MS/MS metabolomics and lipidomics work differently than proteomics, wherein all collected metabolite and lipid fragment spectra must be matched to a spectrum collected in a spectral library to confidentially identify a molecule (77). This process thus limits the breadth of information that is obtainable from such an experiment and requires in-depth and intensive metabolite library construction or the purchase of many different small-molecule standards to increase metabolite or lipid identity confidence.

In 1987, Lin et al. (78) were the first to study the spheroid metabolome using NMR. The authors designed a system to keep MCF7 and V79 spheroids in an NMR tube with perfusion of nutrients to spheroids for continual measurement, observing that perfusion of nutrients caused a sharp increase in ^31^P-ATP signal due to spheroids undergoing metabolism again (78). NMR became popular for studying the metabolome of spheroids, with Santini et al. (79) showing differences in MG63 osteosarcoma spheroids and monolayer culture metabolites in 2004. In 2012, some of the first uses of MS for understanding the spheroid metabolome emerged. Kotze et al. (80) used time-of-flight secondary ion MS (ToF-SIMS) on FaDu squamous cell carcinoma spheroids to show distinct metabolic differences in lipids and phosphates in the core and outer regions of the spheroid.

Advances in the field of MS and bioinformatics has facilitated progress in elucidating spheroid metabolomics. Wen et al. (81) recently studied the metabolomics of U87 glioma spheroids and monolayer cultures. Reduced levels of nucleotide, glutathione, and amino acid metabolism were observed in spheroids compared to monolayer cells. Using ^13^C_6_-labeling fluxomics, the authors showed that spheroids had elevated glycolytic flux compared to the monolayer cultures (81). Research from De Vitis et al. (82) showed variations in specific metabolites from monolayer and spheroid cell cultures of MCF7 and T47D breast cancer cell lines as well as H460 and HCC827 lung adenocarcinoma cell lines. Seven metabolites showed consistent trends between all spheroid and monolayer cultures: downregulation of d-glucose monophosphate, d-hexose pool, uridine diphosphate (UDP)-glucose, d-fructose monophosphate, dIMP, l-aspartic acid, and l-serine in spheroids and upregulation of l-lactic acid in spheroids compared to their corresponding monolayer cultures (82). Lastly, Xie et al. (83) used untargeted LC-MS/MS to determine the differences in metabolites in monolayer and spheroid HepG2 liver cancer cells as well as solid tumors. HepG2 spheroids and solid tumor samples had more similar lipid ions with 900.0 *m*/*z* and above compared with monolayer cells in negative ionization mode, mostly attributed to PI lipids. The same trend was observed in positive ionization mode 650–700 *m*/*z* and 850–900 *m*/*z*, with the latter range being attributed to triacylglycerol lipids (83). Specifically, the authors observed lysophosphatidylethanolamine [LPE (18:0)] to be localized to the spheroid core, while in solid tumors it was largely relegated to the parenchyma of the tissue (83).

**Figure 5:**
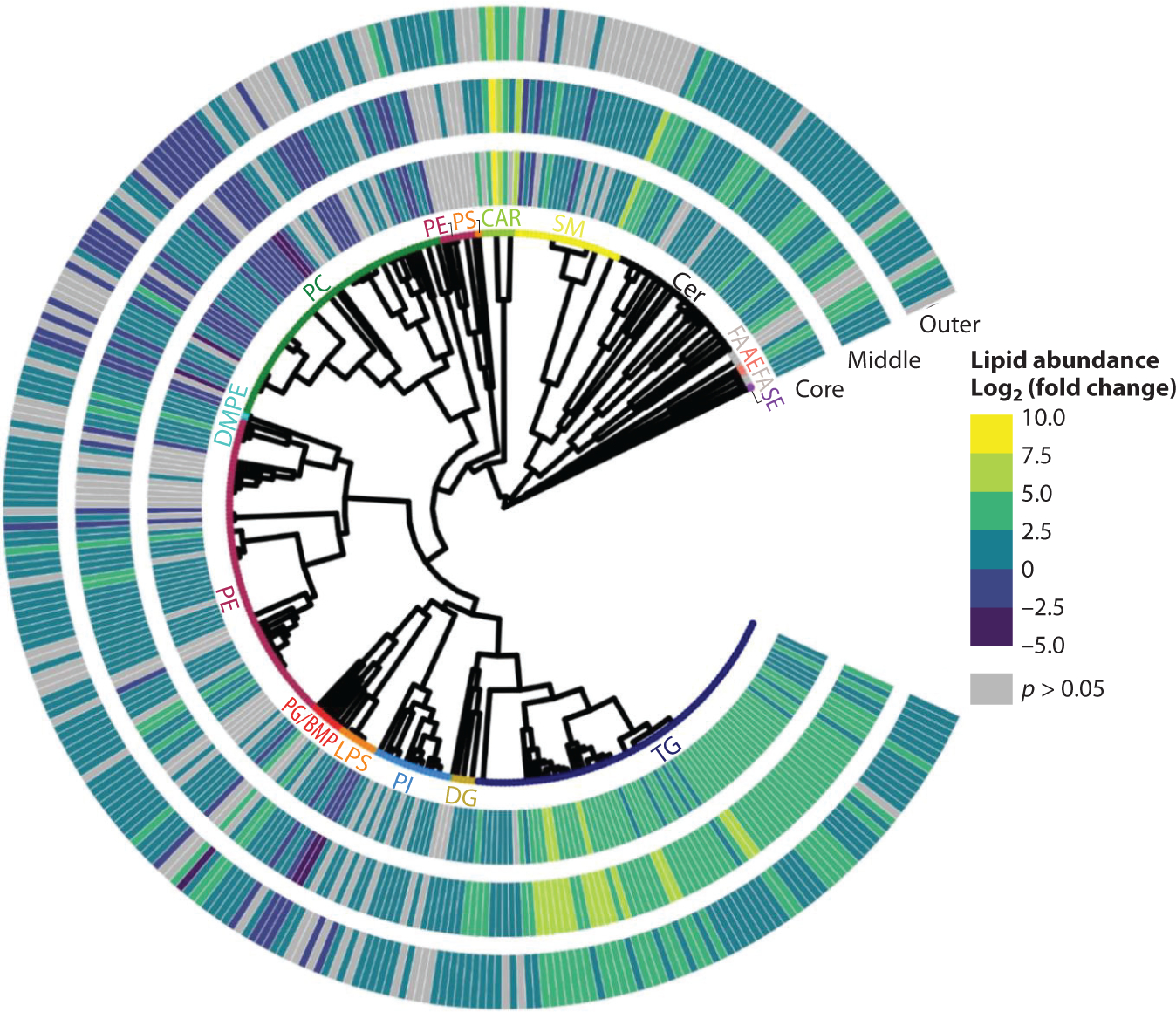
Differentially expressed lipids comparing the lipid abundance found within spheroid layers to the monolayer lipid abundance. Lipids were identified at the tandem mass spectroscopy level and shown and clustered according to their putative three-dimensional structure. The lipid abundance are depicted as log_2_ fold-change ratios between a spheroid layer and a two-dimensional monolayer culture sample. Elevation of TG lipids observed in spheroids as well as a decrease in the abundance of PC lipids. Gray bars indicate *p* > 0.05. Figure adapted with permission from Reference 84; copyright 2021 Wiley. Abbreviations: AE, acylethanolamine; BMP, bis(monoacylglycero)phosphate; CAR, carnitine; Cer, ceramides; DG, diacylglycerol; DMPE, 1,2-dimyristoyl-*sn*-glycero-3-phosphoethanolamine; FA, fatty acid; LPS, lipopolysaccharides; PC, phosphatidylcholine; PE, phosphatidylethanolamine; PG, phosphatidylglycerol; PI, phosphatidylinositol; PS, phosphatidylserine; SE, sphingosine; SM, sphingomyelin; TG, triacylglycerol.

Just as the proteome of the different layers of the spheroid has been characterized, progress has been made to elucidate both the metabolome and lipidome of the regions of the spheroid. A study from Xia et al. (9) used nanocapillaries to extract the outer proliferative layer, inner necrotic core, and the region closest to the tissue culture surface (also termed the lower region) of the spheroid, showing distinct metabolic differences in each region. Pyrimidine metabolites were found to be lower in abundance in the inner necrotic region compared to the outer regions, and pyrimidine metabolism was elevated in the upper regions of the spheroid compared to the lower region (9). Only three metabolites were more abundant in the lower region of the spheroids: purine, taurine, and proline (9). Another study conducted by Tobias & Hummon (84) used serial trypsinization to peel away the layers of HCT 116 spheroids and conduct untargeted lipidomics on these layers (84). There were significantly (*p* < 0.0005) higher levels of triacylglycerol lipids in all three spheroid layers compared to monolayers, with the middle layer having the highest amount of triacylglycerol lipids. Alterations to many other lipid species were observed, with many decreases to phosphatidylcholine (PC) and phosphatidylethanolamine (PE) lipids (84) (Figure 5).

The metabolome of spheroids has also been explored spatially through MSI to show the distribution of metabolites through different regions of the spheroid. Using MSI, Zang et al. (85) showed that various metabolites such as xanthine, hypoxanthine, and inosine were more abundant in the outer regions of an esophageal tumor spheroid. The authors also observed decreasing intensities of PC(32:1), PC(34:1), PE(34:1), PE(36:1), glycerophosphocholine (GPC), and glycerophosphoethanolamine (GPE) as spheroids were developing from days two through seven (85). A recent study by Chen et al. (86) used MALDI MSI to elucidate the spatial metabolome of MDA-MB-231 triple-negative breast cancer cells cocultured with Jurkat T cells. Many metabolic differences were observed between monoculture and cocultured spheroids, such as elevated levels of taurine, glutamate, phosphocholine, phosphoethanolamine, and C14–C16 long-chain fatty acids in cocultured spheroids. By stimulating the immune response of the Jurkat T cells, the authors observed upregulation of malic acid, glutamate, glutathione, and phenylalanine along with downregulation of various lipid species, such as PC(34:1) (86).

Metabolite identification can hinder the utility of an MSI experiment, as only an MS1 spectrum is obtained for an MSI experiment, and it is challenging to confidentially identify a metabolite using only an MS1 signal. A method to identify these metabolites in an MSI experiment is to conduct untargeted MS/MS metabolomics on samples used for MSI. Xie et al. (87) used both MSI to spatially map metabolites of bisphenol S (BPS; a pollutant commonly used in consumer plastic products) in MDA-MB-231 breast cancer spheroids and untargeted LC-MS/MS for metabolite identification and quantification from these spheroids. The authors observed that BPS is able to fully penetrate the spheroid within 4 h and that there were elevated levels of tricarboxylic acid cycle metabolites in BPS-treated spheroids. Xie et al. then mapped altered metabolites found in the metabolomics analysis to the MSI data and observed elevated signal intensities of adenosine triphosphate (ATP), adenosine diphosphate (ADP), adenosine monophosphate (AMP), and guanidine monophosphate (GMP) in BPS-treated spheroids primarily in the outer proliferative layer of the spheroid (87).

Metabolomics and lipidomics can complete the picture of how a cancer spheroid will respond to various therapeutics, allowing one to determine whether there are any undesired or off-target effects. Recently, Fries et al. (88) studied the lipidome of HCT 116 and HT-29 colorectal cancer spheroids inhibited with either a generation 1 (cerulenin) or 2 (TVB-2640) fatty acid synthase inhibitor using untargeted LC-MS/MS lipidomics. The authors observed remodeling of the cellular lipidome, with many alterations to triacylglycerol and glycerophospholipids. The authors also observed increases in polyunsaturated fatty acid (PUFA) lipid levels in the generation 2–inhibited spheroids and decreased PUFA levels in generation 1–inhibited spheroids (88). In another study, Li et al. (89) characterized the penetration of amiodarone in HepG2 spheroids as well as the spatial metabolic changes in the spheroids after amiodarone treatment using MSI. Amiodarone caused significant downregulation of many fatty acids and PC lipids and caused upregulation of PI and phosphatidylglycerol (PG) lipids (89).

## ALTERNATIVE MODELS

Although spheroids have become a well-studied model and show great promise for a better understanding of pharmaceuticals, more intricate cell culture models have also been developed. One such model previously mentioned, organoids, are one of these more complex models. Organoids have been further reviewed by Kim et al. (90) and Tang et al. (91). Similar to spheroids, organoids adhere to each other in a 3D manner and are often spherical. However, organoids are typically hollow and develop microstructures that resemble the organ of origin (92, 93). Organoids, though, require more advanced cell culture techniques with more specialized and expensive reagents as well as longer growth times, reducing the throughput of this system. One of the immense benefits of organoids is the ability to derive them from patients, allowing for these models to be used for personalized medicine purposes. Cristobal et al. (94) studied the proteome of healthy and cancerous colon organoids derived from seven different patients to show personalized differences among the patient proteomes. The authors showed distinct patient-specific features for both the tumor and healthy organoids, such as downregulation of the MSH3 protein in one patient and no change in this protein in the other six patients. This discovery was a vital finding, as this one patient may be classified as having a microsatellite instable tumor, which thus allows for specific treatment options to potentially improve patient survival (94).

Organ-on-a-chip is another popular complex cell culture model that has been further reviewed by Leung et al. (95). This cell culture system can more accurately represent the functions and properties of an in vivo organ by coculturing with multiple cell types and microfluidic channels to deliver nutrients/therapeutics and remove metabolic waste. Just like organoids, organ-on-a-chip requires more advanced cell culture techniques than spheroids. Organ-on-a-chip studies require delicately fabricated microfluidic devices that can be challenging to maintain on a high-throughput scale. Though complicated, organ-on-a-chip allows modeling of complex and vital cellular interactions without the need for an animal model. In 2020, Xu et al. (96) demonstrated an organ-on-a-chip system to model lung cancer–derived brain metastasis (BM). The authors cocultured epithelial and endothelial cells on opposite sides of each other in a microfluidic device to have artificial healthy lungs for air flow and a vasculature for nutrient delivery, respectively. The authors added PC9 lung adenocarcinoma cells to the lung half of the microfluidic device to allow the cells to extravasate to a neighboring chamber containing brain astrocyte cells. Comparing isolated BM PC9 cells with primary PC9 cells, the authors observed upregulation of aldehyde dehydrogenases and that glutathione metabolism was elevated in BM PC9 cells. These proteomic results on this advanced model have helped glean insights into the acquired resistance observed in patient lung cancer brain metastasis (96). There has also been recent progress to develop a spheroid- and/or organoid-on-a-chip (97, 98) and to coculture endothelial cells with spheroids to generate a vasculature (99).

There is active development of other 3D cell cultures that circumvent some of the challenges posed by spheroids. The first challenge is that not every cell line is able to make freestanding spheroids because some cell lines have little de novo synthesis of scaffolding proteins. Recent research by the Lockett group focused on stacking paper-based cell cultures to rapidly synthesize spheroids from many cell types regardless of their ability to make a freestanding spheroid because the cells are suspended in a hydrogel (100–103). Another challenge is the time it takes to allow spheroids to fully develop and maintain, possibly reducing the throughput of this model compared to the conventional monolayer cultures. Commercially available systems under development automatically dispense spheroids into wells for analysis, allowing for higher-throughput analysis of therapeutics and conditions in spheroids (104).

## CONCLUSION

With the passage of the FDA Modernization ACT 2.0, it is imperative that more analytical techniques are developed to analyze complex cell culture systems (24). As discussed in this review, cancer spheroids are a common cell culture system with a higher level of complexity than their monolayer counterparts, while still allowing the possibility of high-throughput screening. Cancer spheroids develop distinct cellular layers that more accurately mimic an avascularized tumor. Various pharmaceuticals have been shown to interact within the spheroid model system differently than the conventional monolayer system used for therapeutic testing. Other more technically challenging—but more advanced—cell culture models exist for recapitulating an in vivo tumor or organ better than a spheroid. The future of cancer research will be propelled by further development of the analytical techniques to characterize the complex biology found within spheroids and other complex cell culture model systems.

## Figures and Tables

**Table 1:** Summary of chemotherapeutics that have been studied in cancer spheroids

Chemotherapeutic	Disease	Technique	Reference(s)
Amiodarone	Liver cancer	MSI, metabolomics	89
Cerulenin	Colorectal cancer	MSI, lipidomics	88
Cetuximab	Colorectal cancer	MSI	41
Cisplatin	Ovarian cancer	Genomics	32
Doxorubicin	Ovarian cancer	Genomics	32
Ertredin	Liver cancer	Proteomics	66
Hydroxychloroquine	Lung cancer	MSI, lipidomics	37
Irinotecan	Colon cancer	MSI	4, 40
Lapatinib	Breast cancer	Phosphoproteomics	55
Methotrexate	Osteosarcoma, ovarian cancer	Genomics	29, 32
Paclitaxel	Colon cancer, ovarian cancer	Genomics	31, 32
Regorafenib	Colon cancer	Proteomics	62
Temozolomide	Glioblastoma	Proteomics	67
Topotecan	Ovarian cancer	Genomics	32
TVB-2640	Colon cancer	MSI, lipidomics	88
Vincristine	Ovarian cancer	Genomics	32

Abbreviation: MSI, mass spectrometry imaging.
